# The Effect of Three Irrigants on the Coronal Leakage of the Root Canals System Irrigants

**Published:** 2010-08-15

**Authors:** Maryam Zare Jahromi, Mehrdad Barekatain, Maziar Ebrahimi, Bahare Askari

**Affiliations:** 1. Department of Endodontics, Dental School, Islamic Azad University of Khorasgan, Isfahan, Iran.; 2. Department of Operative Dentistry, Dental School, Islamic Azad University of Khorasgan, Isfahan, Iran.; 3. Dentist, Isfehan, Iran.

**Keywords:** Citric Acid, EDTA/NaOCl, Irrigation, Microleakage, MTAD, Smear Layer

## Abstract

**INTRODUCTION:**

The production of smear layer during canal instrumentation is thought to increase coronal microleakage even after canal obturation. Previous studies have shown that the type of irrigant does not necessarily affect the seal of the obturation. Our study aimed to evaluate the effect of three irrigation solutions (MTAD, citric acid and EDTA/NaOCl) on the coronal microleakage of root canals.

**MATERIALS AND METHODS:**

Fifty five intact single rooted teeth were instrumented and randomly divided into three experimental groups (15 teeth each) and two control groups (5 teeth each). Final irrigation was carried out with MTAD in group I, citric acid in group II, and EDTA/NaOCl in group III. EDTA/NaOCl was used for the negative control group and saline irrigation was carried out in the positive control group. After lateral compaction with gutta-percha, the access cavities of the experimental specimens were restored with temporary restorative material. Temporary cement was not used in the positive control group. In the negative control group, access cavities and foramen apices were sealed with glass ionomer. Microleakage of samples was measured using the dye penetration technique. Data were analyzed with ANOVA and Tukey test to determine statistical differences between groups.

**RESULTS:**

MTAD, citric acid and EDTA/NaOCl all had less microleakage compared to normal saline. However, no difference was detected between the experimental groups.

**CONCLUSION:**

In this study, all three groups demonstrated effective seal with gutta-percha obturation. This is likely to be due to various factors including their ability to remove smear layer.

## INTRODUCTION

Endodontic treatment success has been historically based on effective debridement, disinfection, and obturation [1]. Many researchers have stressed the effect of coronal seal on the prognosis of root canal treatment in preventing bacterial penetration [2][3][4][5].

Smear layer is a combination of organic and inorganic components formed during root canal preparation. This layer consists of dentin debris, pulp remnants, bacteria, endotoxin and sometimes restorative materials [5]. It seems that removal of smear layer after root canal instrumentation and before canal obturation improves adaptation of root filling materials to the canal walls, resulting in a superior seal and more predictable outcomes [6][7].

Citric acids, phosphoric acid, sodium hypochlorite, EDTA, EDTAC and Carbamide Peroxide have all been used to remove the smear layer [8]. MTAD has been introduced as an irrigant. It has good antibacterial properties and great potential for removing the smear layer [9].

This type of irrigant was found to positively reduce coronal microleakage. In addition, combining irrigants with smear layer removing etchants such as EDTA with NaOCl, and chlorhexidine gel provided better coronal seal when compared with NaOCl and chlorhexidine or distilled water alone [10].

Park et al. showed that the combination of MTAD and NaOCl effectively removed smear layer and significantly reduced coronal leakage. However, no statistically difference was detected between MTAD and NaOCl/EDTA [11].

A recent study has illustrated that there is no difference in the smear layer removal efficacy between 17% EDTA, 10% citric acid and Smear Clear. However, 10% citric acid was less effectiveness in apical third compared to coronal and middle third. None of the materials used in the study were able to completely remove the smear layer [12].

Ghoddusi et al. demonstrated that teeth treated with 17% EDTA or MTAD had no differences in bacterial penetration and microleakage. However, leakage occurred in a prolonged duration compared to teeth irrigated with NaOCl [13].

Therefore this study aimed to compare the coronal leakage of teeth treated with EDTA/NaOCl, MTAD or citric acid.

## MATERIALS AND METHODS

Fifty five human single rooted teeth with intact crown and root that did not have calcification or severe root curvatures, were selected. Teeth were randomly divided into three groups of 15 samples, i.e. groups I was irrigated with MTAD, group II with 10% citric acid, and group III with 5.25% NaOCl and 17% EDTA. Ten samples were equally divided between negative and positive control groups (5 each).

All specimens were cleaned using amalgam polishing burs and disinfected in 5.25% sodium hypochlorite (Daroogar, Tehran, Iran) for 20 min. All samples were stored in a 0.9% normal saline solution until used.

After access cavity preparation, root canals were prepared with crown-down technique using K-files (Mani, Astonomia, Japan). The canals were instrumented to a size #40 master apical file (MAF) 1 mm short of the apex. Canal flaring was performed with number 1, 2, and 3 Gates Glidden drills followed by number 1, 2, 3 Peeso reamers. After two files were used canals were irrigated with 2 mL of 5.25% NaOCl throughout the procedure. After instrumentation, radiographs were taken with the MAF in place to ensure proper length and positioning within the canal.

Before obturation, canals in each experimental group were irrigated with 5 mL of the specific irrigant to remove the smear layer. The canals were gently dried with paper points. After that, radiographs were taken with the master cone in place to ensure proper length and positioning within the canal. The canals were laterally condensed with gutta-percha and AH26 sealer (Roth International Ltd., Chicago, IL) using finger spreader. The remnant gutta-percha was removed 2 mm below the cementoenamel junction with a hot excavator and then vertical condensation was performed. All the procedures were conducted by one operator.

After obturation, radiographs were taken to check the quality and length of the obturation. Access cavities were sealed with a Zonalin temporary cement (Golchai, Tehran, Iran). Finally samples were covered with three layer of nail varnish from CEJ to the apex.

In the positive control group, saline was used for final irrigation. The root canals were obturated as the experimental groups; however, temporary restoration was not used. Finally, outer surfaces were covered with three layer of nail varnish.

In negative control group final irrigation was performed with hypochlorite sodium followed by EDTA. Then, the canals were obturated by lateral compaction using gutta-percha and AH26 sealer. The access cavity, foramen apical and surrounding areas of each specimen was sealed with glass ionomer. Subsequently, all teeth surfaces (including crown and root) were covered with three layer of nail varnish.

After two weeks storage in 37ºC saline, each sample was placed with the crown facing the top of the tube in a test tube filled with methylene blue (Merck, Darmstadt, Germany). Tubes were centrifuged at 12000 rpm for 20 min. Then vertical buccolingual section was made using a diamond disc cutting in nonstop device (Bego, Berman, Germany).

The leakage of samples was measured with a stereomicroscope (100B, Loma, Russia). Dye leakage mean was measured at two sides of the canal walls. Data were analyzed with ANOVA test and pairwise comparisons of groups were performed by post hoc Tukey tests.

## RESULTS

The mean microleakage and standard deviation of different groups are shown in Table 1. ANOVA test results showed that significant differences exist between groups (P<0.001). Further analysis for pairwise comparison using post hoc Tukey tests showed no significant differences between experimental groups (P>0.05). However, MTAD, citric acid and EDTA/NaOCl groups had significant differences with both positive and negative control groups (P<0.05).

**Table 1:** Distribution of microleakage in apical of root canals

**Group**	**Number of** ** Specimen**	**Mean** ** of leakage**	**Standard** ** Deviation**	**Min.** ** of leakage**	**Max.** ** of leakage**	**Result**
10% citric acid	15	10.64	3.99	4.64	20.26	P=0.4
MTAD	15	9.87	2.55	5.52	15.13	
17% EDTA + 5.25% NaOCl	15	8.90	2.72	4.04	16.41	

## DISCUSSION

A hermetic seal of the root canal system is a key factor in determining the long-term prognosis of endodontic treatment [1]. Smear layer formed during cleaning and shaping consists of dentinal and bacterial remnants, which may interfere with tight adaptation of filling materials with root canal walls. Therefore, some investigators believe that in the absence of smear layer, a tight contact forms between root canal obturation materials and dentinal wall which improves the seal of the root canal system [2][3][4][5][6][7].

It has been shown some materials such as EDTA and NaOCl, citric acid, phosphoric acid, EDTAC and carbamide peroxide and a newly introduced material, MTAD (Biopure country state), have the ability to remove the smear layer [8][9].

An ideal irrigants should be able to eliminate smear layer. Chelating agent solutions such as EDTA decalcify and soften dentin eliminating the smear layer. EDTA is often used with NaOCl to remove the smear layer. EDTA only removes the inorganic component of smear layer; therefore a proteolytic agent like NaOCl is utlisized for dissolving inorganic tissue. Citric acid is a chelator commonly uses in concentrations of 10%. However, concentrations from 30% to 50% have also been suggested for irrigation and softening of dentine [9].

MTAD is another irrigant which contains tetracycline (doxycycline) as an antimicrobial agent, citric acid for removing the smear layer and a detergent (tween 80) which eases the penetration of solution into the dentinal tubules and canal irregularities. MTAD cannot only kill Enterococcus faecalis but it also has the potential to remove most parts of smear layer although some of organic component of the layer remain on the surface of the canal walls [11]. Using low concentration of NaOCl before application of MTAD enhances the effectiveness of MTAD. Some reports suggest that, compared with NaOCl, MTAD has more antibacterial effect, though others have shown NaOCl to be more effective broad spectrum antimicrobial [1][9].

In the present study we used EDTA, NaOCl and citric acid as they are readily available and have the ability to remove smear layer.

Dye penetration method was conducted as studies have shown that dye penetration is as reliable as bacterial penetration for assessing the sealing ability of a material [14].

Despite the reduction in mean values of coronal microleakage from groups I, II, to III, the differences between the groups were not statistically significant. These findings are comparable to several similar previous studies [11].

Ghoddusi et al. showed that the duration of bacterial penetration in teeth treated with 17% EDTA or MTAD was not significantly different [13]. Khedamt and Shokouhinejad demonstrated that there were no statistical difference in the efficacy of 17% EDTA and 10% citric acid in removing the smear layer [12]. Further study found no differences in coronal leakage between MTAD and EDTA [11]. In the present study, irrigation with 17% EDTA followed by 5.25% NaOCl showed less microleakage compared to MTAD; though not significantly different. These findings may result from the inability of MTAD to remove the organic components of smear layer [11].

In contrast, Torabinejad et al. used MTAD in the canal after irrigation with NaOCl, distilled water or 17% EDTA. They showed MTAD was able to remove most parts of smear layer but some organic component of the layer remained in the canal [9].

Furthermore, some differences with other investigations may be due to the variety in materials and the methods. Overall, all experimental groups showed significantly lower coronal microleakage compared to positive control group.

## CONCLUSION

Irrigating the root canal system with solutions that can dissolve the smear layer, such as MTAD, citric acid, and EDTA (with NaOCl) will reduce coronal microleakage from the obturated canal more efficiently than saline. Interestingly, none of the irrigants were found to be significantly superior.
